# Role of artificial intelligence in teledentistry diagnosis and treatment planning

**DOI:** 10.6026/973206300221974

**Published:** 2026-04-30

**Authors:** M Aarti Rajambigai1, Faraz Ahmed, S Mohammed Harris, Jyotirmayee Rath, Megha Varghese, Poorani A Elango

**Affiliations:** 1 Department of Prosthodontics, Rajas Dental College and Hospital, Kavalkinaru, Tamil Nadu, India; 2 Department of Conservative Dentistry & Endodontics, Oral and Maxillofacial Surgery, House surgeon, Burdwan Dental College and Hospital, Khosbagan, Bardhaman, West Bengal, India; 3 Department of Periodontics, SRM Dental College, Bharathi Salai, Ramapuram, Chennai-98, Tamil Nadu, India; 4 Department of Endodontics and Conservative Dentistry, Hitech Dental College, Pandara, Bhubaneswar-751025, Odisha, India; 5 Department of Periodontology, FAME (Farookh Academy of Medical Education), Near Ilavala, Hunsur Taluk, Mysore, Karnataka, India; 6 Department of Periodontology, SRM Dental College, Bharathi Salai, Ramapuram, Chennai, Tamil Nadu, India

**Keywords:** Teledentistry, remote diagnosis, intelligent diagnostic tools, dental AI, digital health

## Abstract

Limited access to dental specialists delays accurate diagnosis and treatment planning in remote and underserved populations. Therefore, it is of interest to evaluate the reliability of an artificial intelligence system for remote dental diagnosis and treatment planning using intraoral images, panoramic radiographs and clinical histories from 320 patients. AI-generated outputs were compared with independent assessments by three board-certified dentists using Cohen's kappa, sensitivity and specificity metrics. The system achieved 93.2% agreement for caries detection, 89.1% for periodontal assessment and 90.5% for malocclusion classification, with caries detection sensitivity of 94.8% and specificity of 92.1%. Thus, use of artificial intelligence showed substantial diagnostic concordance and may improve triage efficiency and access to care in teledentistry settings.

## Background:

Access to dental specialists continues to be a challenge for rural and underserved patients; thus, limiting early identification and thorough treatment planning [1]. To overcome this challenge, teledentistry is now being utilized as a digital option for remote consultations, triage and follow-up and secure transmittal of images and data between dental care providers and patients [2]. The availability of digital x-rays, electronic medical records and cloud technology has made teledentistry a viable option for delivering dental care through telemedicine platforms [3]. However, despite the expansion of digital imaging, electronic health records and cloud technology, the success of remote teledentistry is still contingent upon the availability of clinicians, variations between different interpreters and the accuracy of image interpretation [4]. Artificial intelligence (AI) models using deep learning and computer vision have consistently produced diagnostic results for clinicians regarding dental caries, periodontal bone loss and orthodontic issues from both x-rays and intraoral photographs [5, 6]. With respect to AI's diagnostic capabilities, research indicates that the sensitivity and specificity of AI are comparable to that of trained professionals in highly controlled studies [7]. AI systems may also allow for the automatic tracing of cephalometric lines, arch analysis and developing structured treatment planning [8]. Although there are tremendous advances made by using AI, there are still limited prospective validations of AI performance within the parameters of online teledentistry [9]. Therefore, it is of interest to conduct a complete evaluation of the AI diagnostic and treatment planning system within an established teledentistry model.

## Materials and Methods:

This prospective observational study was conducted using a secure teledentistry platform that collected patient submissions between January and September 2025. A total of 320 patients who submitted intraoral photographs, panoramic radiographs and brief clinical histories were included. Patients with incomplete imaging, poor image quality or prior ongoing dental treatment were excluded. All data were anonymized before analysis. The study protocol followed the principles of the declaration of Helsinki. Informed consent was obtained from all participants prior to data submission. An artificial intelligence system combining a convolutional neural network for image analysis and a natural language processing pipeline for clinical text interpretation generated diagnostic labels and treatment planning suggestions. The model was previously trained using annotated dental imaging datasets and validated internally prior to deployment. Three board-certified dentists independently reviewed each case and provided diagnoses and treatment plans. Reviewers were blinded to AI outputs to avoid assessment bias. Disagreements between clinicians were resolved by consensus. Diagnostic agreement between AI and clinicians was evaluated using sensitivity, specificity, overall accuracy and Cohen's kappa statistics. Confusion matrices were generated for caries detection, periodontitis diagnosis and malocclusion classification. Statistical analysis was performed using SPSS software (version 26.0). A p-value <0.05 was considered statistically significant.

## Results:

A total of 320 patient submissions were analysed using the teledentistry platform. The artificial intelligence system demonstrated high diagnostic agreement with clinician assessments across all evaluated conditions. Agreement was highest for dental caries, followed by malocclusion classification and periodontal disease assessment. Sensitivity and specificity values indicated strong diagnostic reliability. Most discrepancies occurred in early gingivitis and in cases with overlapping restorative conditions. The AI system achieved an overall diagnostic agreement of 93.2% for caries detection. Periodontal health assessment showed 89.1% agreement, while malocclusion classification reached 90.5% concordance with clinician evaluations. Cohen's kappa values indicated near-perfect agreement for caries (0.88), substantial agreement for malocclusion (0.82) and periodontal disease (0.79). Sensitivity for caries detection was 94.8%, with a specificity of 92.1%. The system correctly identified 187 cases of active caries and 108 non-caries cases. Periodontitis detection showed 86.3% sensitivity and 91.5% specificity. The AI correctly detected 113 cases of periodontitis and 179 healthy cases. Malocclusion classification demonstrated 90.5% sensitivity and 91.9% specificity, correctly identifying 46 cases requiring orthodontic intervention. These findings indicate strong agreement between AI-generated outputs and clinician evaluations in remote dental diagnosis and treatment planning. Table 1 (see PDF) demonstrates the diagnostic tool's key performance metrics, outlining its sensitivity, specificity, accuracy and Cohen's kappa values for the detection of dental caries, periodontitis and malocclusion. Table 2 (see PDF) presents the confusion matrix for caries detection, showing the relationship between predicted and actual cases to highlight the system's diagnostic reliability. Table 3 (see PDF) shows the confusion matrix for periodontitis detection, emphasizing the tool's accuracy in correctly identifying diseased and healthy cases in comparison with clinician evaluations. Finally, Table 4 (see PDF) illustrates the confusion matrix for malocclusion classification, indicating the system's effectiveness in distinguishing between patients with and without malocclusion based on predicted and actual outcomes.

## Discussion:

The study found that the use of an artificial intelligence system to provide diagnostic information was highly concordant with the diagnosis made by board-certified dentists when using teledentistry in real-world practices [10]. When comparing the diagnosis of caries, there was a kappa of 0.88 indicating the highest level of agreement, which concurs with several recent studies that have shown similar or higher sensitivity for carious lesions using radiographs and deep learning techniques [11]. The high specificity found in this study will significantly reduce the chances of over- referring and providing unnecessary treatment when dentists are using remote triage systems [12]. In the case of periodontal assessment, the kappa level of 0.79 demonstrates slightly lower agreement, supporting previous literature demonstrating that early inflammatory changes are still difficult for algorithms to determine based only on images [13]. A significant reason behind the reduced sensitivity in identifying early gingivitis may be attributed to the use of static images, as the subtle changes in color and texture of tissues are difficult to detect in static images [14]. When classifying malocclusion, the level of agreement exceeded 90%, consistent with previous studies validating the use of convolutional neural networks for orthodontic classification [15]. These results support the possibility of implementing AI-assisted triage for both rural and urban settings with high patient volumes. The clinical implications of these findings are substantial. AI systems will act as structured decision support tools that will assist with standardizing initial evaluations and decreasing the variability among clinicians. The ability to automate tagging of images and provide suggestions on treatment will decrease the amount of time that the clinician spends thinking about a diagnosis and will improve efficiency in the clinical workflow. Providing the ability to access specialist evaluations through secure teleconsultation will increase access to specialty evaluations without the immediate need for patients to see specialists in person. However, the outputs produced by AI systems should be interpreted under the supervision of a clinician and care should be taken with borderline or complicated restorative cases. The variation in the dataset and the dependence on two-dimensional images will prevent a complete soft tissue evaluation. It is anticipated that future systems will incorporate dynamic imaging inputs and provide multiple centers with an independent validation of the dataset to increase the generalizability and robustness of the algorithms.

## Conclusion:

Integrated artificial intelligence had great alignment and agreement with remote dental diagnostics and treatment planning by clinicians in a teledentistry workflow. The use of AI as a clinical decision support tool with clinician oversight offers opportunities to enhance the efficiency of triage minimize variability in diagnosis and increase equitable access to oral health care.
